# Controllable Two-dimensional Perovskite Crystallization via Water Additive for High-performance Solar Cells

**DOI:** 10.1186/s11671-020-03338-5

**Published:** 2020-05-13

**Authors:** Ziji Liu, Hualin Zheng, Detao Liu, Zhiqing Liang, Wenyao Yang, Hao Chen, Long Ji, Shihao Yuan, Yiding Gu, Shibin Li

**Affiliations:** 1 State Key Laboratory of Electronic Thin Films and Integrated Devices, and School of Optoelectronic Science and Engineering, University of Electronic Science and Technology of China (UESTC), Chengdu, 610054 Sichuan China; 2 Chongqing Engineering Research Center of New Energy Storage Devices and Applications, Chongqing University of Arts and Sciences, Chongqing, 402160 People’s Republic of China

**Keywords:** Two-dimensional perovskite, Crystallization, Additive, Water, Solar cell

## Abstract

Steering the crystallization of two-dimensional (2D) perovskite film is an important strategy to improve the power conversion efficiency (PCE) of 2D perovskite solar cells (PVSCs). In this paper, the deionized water (H_2_O) additive is introduced into the perovskite precursor solution to prepare high-quality 2D perovskite films. The 2D perovskite film treated with 3% H_2_O shows a good surface morphology, increased crystal size, enhanced crystallinity, preferred orientation, and low defect density. The fabricated 2D PVSC with 3% H_2_O exhibits a higher PCE compared with that without H_2_O (12.15% vs 2.29%). Furthermore, the shelf stability of unsealed devices with 3% H_2_O under ambient environment is significantly improved. This work provides a simple method to prepare high-quality 2D perovskite films for efficient and stable 2D PVSCs.

## Introduction

Recently, two-dimensional (2D) layered perovskites have drawn extensive attention due to their enhanced moisture resistance versus their 3D counterparts, such as CH_3_NH_3_PbI_3_ (MAPbI_3_) and HC(NH_2_)_2_PbI_3_ (FAPbI_3_). The 2D perovskite with the formula of A_2_B_*n* − 1_M_*n*_X_3*n* + 1_ (Ruddlesden−Popper phase), where B is MA^+^, FA^+^, or Cs^+^, M is Pb^2+^ or Sn^2+^, X stands for halide anion, *n* refers to the number of planes of the corner-sharing [MX_6_]^4−^ octahedral, can be formed by incorporating organic long-chain ligands A (such as phenethylammonium (PEA^+^) or butylammonium (BA^+^)) in the inorganic framework. These 2D perovskites possess many unique optoelectronic properties, and have been developed for use in both solar cells [1, 2] and light-emitting diodes [3]. However, the exciton binding energy of the layered 2D perovskite is enhanced on account of the dielectric confinement effect between the organic layer and the inorganic framework [4], which substantially limits the exciton dissociation in the electrical field [5]. Meanwhile, the bulky organic ligands would form insulating spacing layers and inhibit charge transport between neighboring inorganic slabs. Thus, the PCE of 2D PVSCs is much lower than that of their 3D counterparts, which has been already above 25% [6].

To obtain high-performance 2D PVSCs, many efforts have been made, including the hot-coasting [7], additive engineering [8–14], composition engineering [15–26], precursor solvent engineering [27–30], interfacial engineering [31–35], and other special treatments [13, 36, 37]. Among these methods, additive engineering is the frequently used method due to its simplicity and effectiveness. Zhang et al. found that the vertically oriented 2D layered perovskite film can be deposited via incorporating ammonium thiocyanate (NH_4_SCN) additive into the perovskite precursor solution [8, 9]. Therefore, the PCE of 2D PVSCs drastically increases from 0.56 to 11.01%. Qing et al. demonstrated that the quality of 2D perovskite film can be improved by a synergistic effect of two additives in the perovskite precursor solution [10]. Consequently, a hysteresis-free 2D PVSCs with a PCE exceeding 12% has been obtained. Yu et al. showed that the film morphology and the charge transportation in perovskites can be effectively controlled through adding both ammonium chloride (NH_4_Cl) additive and dimethyl sulfoxide (DMSO) solvent into the precursor solution and a PCE of 13.41% was achieved [11]. Fu et al. reported an efficient 2D PVSCs processed with NH_4_SCN and NH_4_Cl additives, yielding an optimal PCE of 14.1% [12]. In our previous work, we found that DMSO and thiosemicarbazide (TSC) exhibit a synergistic effect in improving the morphology, crystallization, and orientation of 2D perovskite films [14]. It is speculated that both DMSO and TSC are Lewis bases [38], which regulate the crystallization process of 2D perovskite through coordination with the perovskite precursor components. As a result, the efficient and stable 2D PVSCs with a champion PCE of 14.15% were obtained.

In the Lewis acid−base concept, a water molecule is an oxygen donor Lewis base that can bond with the lead iodide (PbI_2_) Lewis acid. Meanwhile, the physical and chemical thermodynamic properties of water molecules, like boiling point, solubility, and vapor pressure, are different from frequently-used N,N-dimethylformamide (DMF) solvent. A series of studies have revealed that the water added into the perovskite precursor solution can control the 3D perovskite crystallization, leading to a better photovoltaic performance [39–44]. However, as we all know, using H_2_O as an additive in 2D PVSC has not been reported yet so far.

In this study, water molecules as additive were introduced into perovskite precursor solutions to control the crystallization of 2D perovskite film. The 2D perovskite film (BA_2_MA_3_Pb_4_I_13_, *n* = 4) treated with a suitable amount of water shows good film morphology, enhanced crystallinity and increased orientation ordering. This high-quality 2D perovskite film contributes to the lower trap-state density and then higher photovoltaic performance of 2D PVSCs. The PCE of 2D PVSCs has been improved from 2.29 to 12.15%. More interestingly, water additive based devices exhibit obviously improved shelf stability.

## Method

### Materials

Methyl-ammonium iodide (MAI), PbI_2_, PEDOT:PSS (4083) aqueous solution, n-butylammonium iodide (BAI), phenyl-C61-butyric acid methyl ester (PC_61_BM), spiro-MeOTAD (2,29,7,79-tetrakis(N,N-di-p-methoxyphenylamine)-9,9-spirobifluorene), 4-tert-butylpyridine, lithium bis (trifluoromethylsulphonyl) imide, and bathocuproine (BCP) were ordered from Xi’an Polymer Light Technology Cory. DMF, chlorobenzene, and acetonitrile were purchased from Sigma-Aldrich. Isopropanol was purchased from You Xuan Tech. All reagents and solvents were used directly without further purification.

### Precursor Solution

The pristine BA_2_MA_3_Pb_4_I_13_ precursor solution (0.85 M) was prepared by mixing BAI, MAI, PbI_2_ with a molar ratio of 0.5: 0.75: 1 in DMF. The precursors with various amounts of deionized water were prepared by adding different volume ratios of deionized water into the pristine precursor solution.

### Device Fabrication

The indium tin oxide (ITO) substrates were ultrasonically washed with detergent, acetone, absolute ethyl alcohol, and deionized water in succession, followed by a 15 min UV-ozone treatment. For the hole collection layers, PEDOT:PSS aqueous solution was spin-coated onto the cleaned ITO substrates at 4000 rpm for 40 s. After the spin-coating, the PEDOT:PSS films were heated in air at 150 °C for 15 min, and then transferred into the glovebox. For the photoelectric conversion layers, the ITO/PEDOT:PSS substrates were preheated at 100 °C for 3 min, followed by spin coating different perovskite precursor solutions at 5000 rpm for 25 s and then annealing at 100 °C for 10 min. For the electron extraction layers, the solution of PC_61_BM (15 mg/mL in chlorobenzene) was spin-coated onto the perovskite layers at 2000 rpm for 30 s. Next, BCP in isopropanol with a concentration of 0.8 mg/ml was spin-coated at 5000 rpm for 30 s. Finally, 70 nm Ag electrodes were thermally evaporated on the BCP layers through the shadow masks. The effective device area was 0.04 cm^2^. For the preparation of hole-only devices, the spiro-OMeTAD layers were deposited onto the 2D perovskite/PEDOT:PSS/ITO substrates by spin-coating spiro-OMeTAD solution at 4000 rpm for 30 s followed by evaporation of 70 nm gold electrode on the top of the device. The spiro-OMeTAD solution was prepared by dissolving 90 mg spiro-OMeTAD, 22 μL of a stock solution of 520 mg/mL lithium bis(trifluoromethylsulphonyl)imide in acetonitrile and 36 μL 4-tert-butylpyridine in 1 mL chlorobenzene.

### Measurement and Characterization

The current density-voltage (*J-V*) curves of PVSCs were measured by Keithley source unit 2400 under AM 1.5G sun intensity illumination by a solar simulator from Newport Corp. The scanning rate of J-V curves is 0.2 V/s. Scanning electron microscope (SEM) measurements were conducted on field emission fitting SEM (FEI-Inspect F50, Holland). The grazing incidence wide-angle X-ray scatting (GIWAXS) measurements were conducted at BL14B1 beamline at Shanghai Synchrotron Radiation Facility, Shanghai, China, with a 0.6887 Å primary beam, 0.2° incident angle. The absorption spectrum of 2D perovskite was measured using Shimadzu 1500 spectrophotometer. External quantum efficiencies were measured by QTEST HIFINITY 5 (Crowntech). Time-resolved photoluminescence spectrum was performed with a Fluo Time 300 (Pico Quant) spectrofluorometer.

## Results and Discussion

**Fig. 1:**
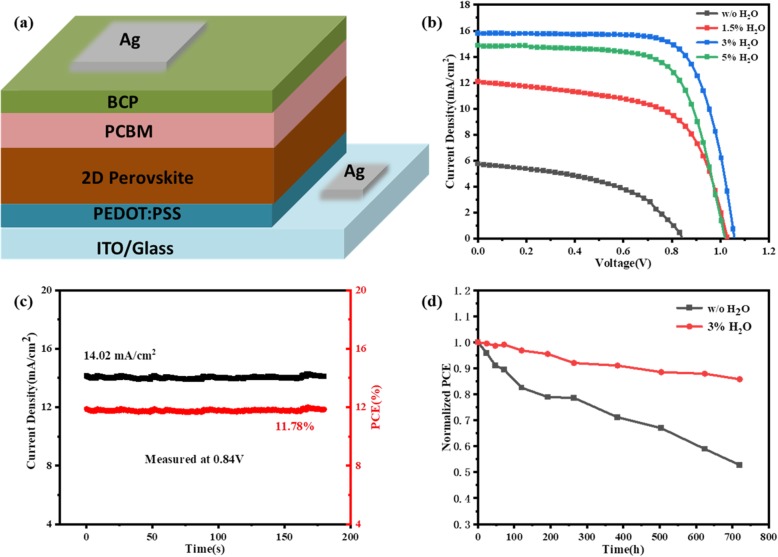
**a** Schematic illustration of PVSC structure. **b** J-V curves of PVSCs based on BA_2_MA_3_Pb_4_I_13_ films deposited from perovskite precursor solutions doped with different volume H_2_O. **c** Steady-state photocurrent and PCE of the champion device 1 sun condition. **d** Long-term stability of the unsealed device without and with 3% H_2_O

To investigate the influence of H_2_O additive on the performance of 2D PVSCs, we fabricated the inverted devices with the configuration of indium tin oxide (ITO)/PEDOT:PSS/BA_2_MA_3_Pb_4_I_13_/PC_61_BM/BCP/Ag as shown in Fig. 1a. The deionized water was mixed with perovskite precursor solution with a varied volume ratio from 0 to 5%. The photocurrent density–voltage (*J–V)* curves of the champion 2D PVSCs based on perovskite with various amounts of water additive under illumination of AM 1.5G, 100 mW/cm^2^ are shown in Fig. 1b, and the corresponding photovoltaic parameters are listed in Table 1. The control device without water additive exhibits a low open-circuit voltage (*V*_*oc*_) of 0.84 V, a short-circuit current density (*J*_*sc*_) of 5.73 mA/cm^2^, a fill factor (*FF*) of 47.63 %, resulting in a poor *PCE* of 2.29 %. From Table 1, it is clear that the suitable amount of H_2_O additive improves the corresponding photovoltaic performance of the devices dramatically. In the case of 2D perovskite with 3% H_2_O, the best-performing device shows a *PCE* of 12.15 %, with a *V*_*oc*_ of 1.06 V, *J*_*sc*_ of 15.80 mA/cm^2^, and *FF* of 72.56 %. The significant improvement in PCE is attributed to the additive-treated perovskite film, which shows a higher crystallinity, larger brick-like grains, uniform morphology, and vertical-orientation perpendicular to the substrate. The details will be discussed below. By further increasing the volume ratio of H_2_O to 5%, the photovoltaic parameters of PVSCs were deteriorated. Figure 1c presents the steady-state photocurrent density where PCE is a function of time at the maximum power point (0.84 V). The PCE of the champion device with 3% H_2_O stabilizes at 11.78% (black) with a photocurrent density of 14.02 mA/cm^2^ (red) in the scan time of 200 s, and it is close to the value extracted from *J-V* curve. Importantly, the shelf stability is one of the key requirements for practical application of PVSCs. Both the unsealed devices without and with 3% H_2_O were stored in air atmosphere with relative humidity of 25 ± 5% at 25 °C to examined evolution of their PCE as a function of time. As shown in Fig. 1d, the device with 3% H_2_O still retained 85.76 % of its initial PCE after 720 h, which was much stable than that of the device without H_2_O (52.76 %). The significantly improved stability is attributed to the stable hydrated 2D perovskites that may be generated during the spin-coating and annealing process. The stable hydrated 2D perovskites resist the decomposition of 2D perovskite film to some extent [39, 40]. On the basis of above results, we conclude that the device treated with optimal water content not only yields superior photovoltaic performance but also shows a good stability.

**Table 1:** The photovoltaic parameters of the champion PVSCs based on perovskite precursor solution with and without water additive

Doping ratio	*V*_*oc*_ (V)	*J*_*sc*_ (mA/cm^2^)	*FF* (%)	*PCE*_*ma*x_ (%)	*PCE*_*avg*_ (%)
w/o H_2_O	0.84	5.73	47.63	2.29	1.85
1.5% H_2_O	1.03	12.07	61.40	7.63	6.59
3% H_2_O	1.06	15.80	72.56	12.15	11.38
5% H_2_O	1.02	14.88	68.38	10.38	9.02

**Fig. 2:**
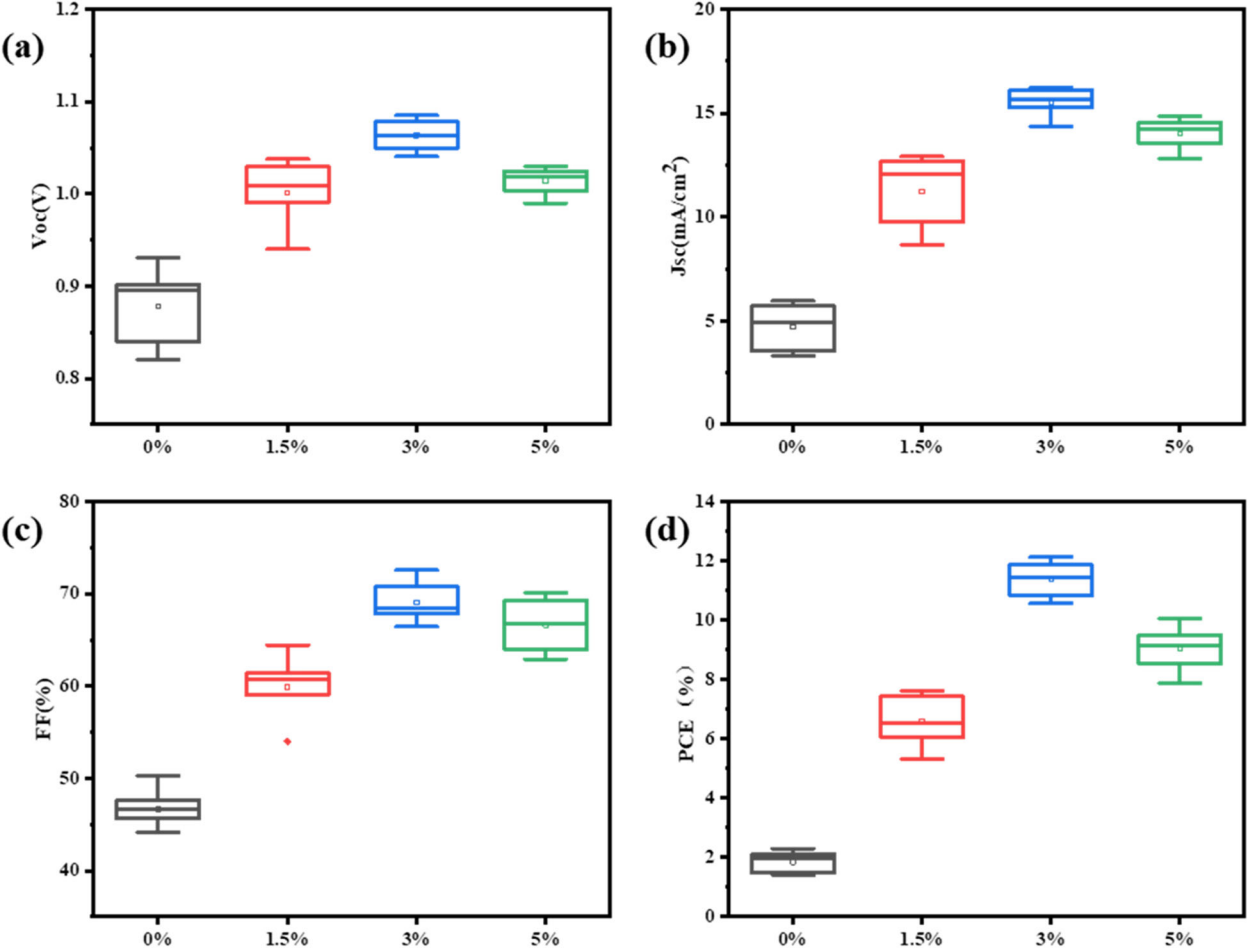
Statistic distribution for (**a**) *V*_*oc*_, (**b**) *J*_*sc*_, (**c**) *FF*, and (**d**) *PCE* of 2D PVSCs based on BA_2_MA_3_Pb_4_I_13_ films with various amounts of H_2_O additive

The statistical data for photovoltaic parameters of 16 PVSCs in each case are shown in Fig. 2a–d. The devices without and with 1.5%, 3%, and 5% H_2_O present the best PCE of 2.29%, 7.63%, 12.15%, and 10.38% with the average value of 1.85%, 6.59%, 11.38%, and 9.02%, respectively (Table 1). These statistical data show the same tends as their corresponding champion devices, proving the statistically meaningful performance improvements of the device upon a suitable amount of deionized water.

**Fig. 3:**
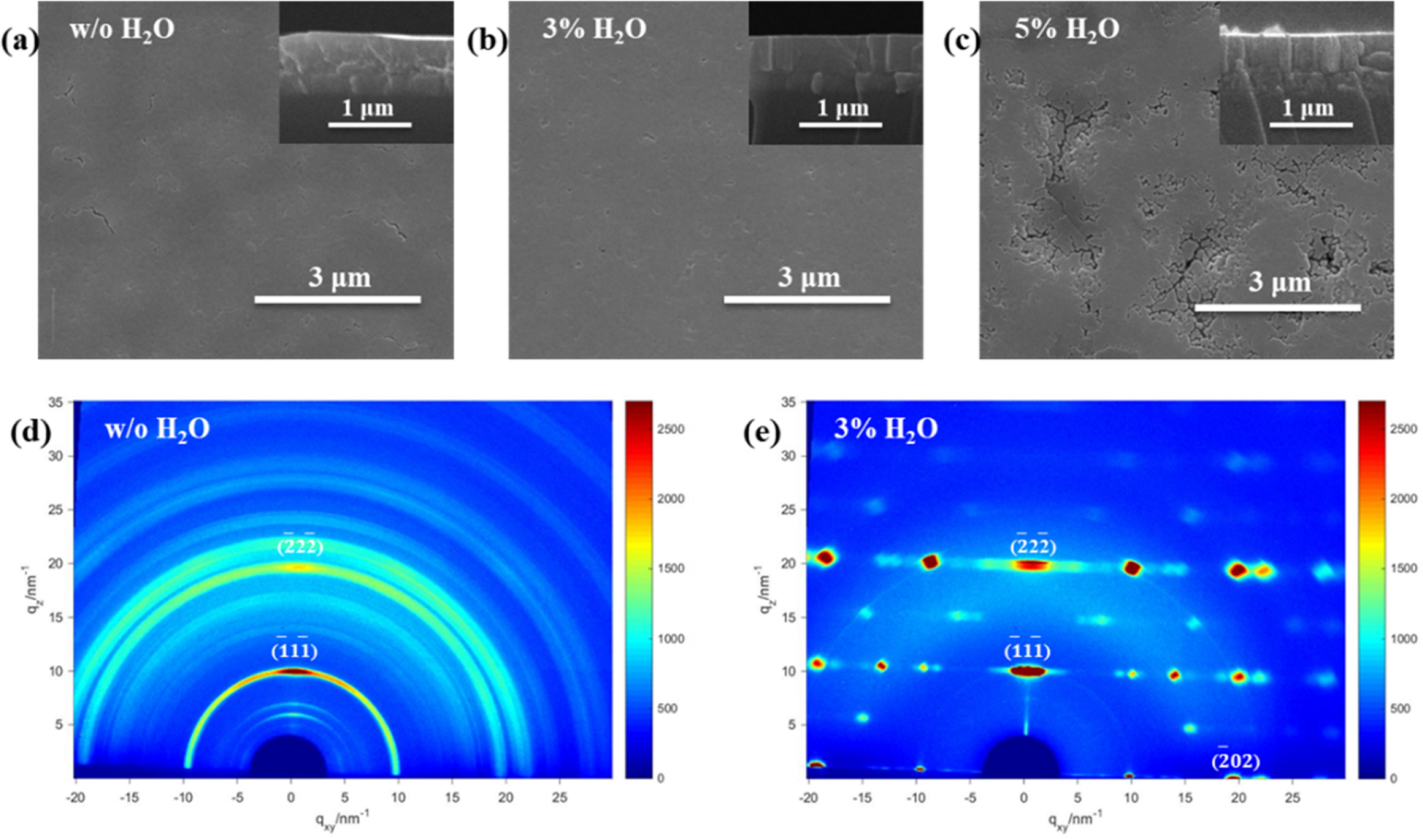
**a**-**c** Top-view SEM images and cross-section SEM images (insets) of BA_2_MA_3_Pb_4_I_13_ films with various amounts of H_2_O additive. GIWAXS patterns of BA_2_MA_3_Pb_4_I_13_ film: (**d**) without H_2_O additive and (**e**) with 3% H_2_O additive

The SEM was conducted to evaluate the effects of H_2_O additive on morphology and coverage of 2D perovskite films. The top-view SEM images of BA_2_MA_3_Pb_4_I_13_ film with various amounts of H_2_O additive are shown in Fig. 3a-c, and the corresponding cross-section SEM images are shown in the insets of Fig. 3a-c. The perovskite film without H_2_O (denoted as perovskite-w/o H_2_O) exhibits a poor morphology with small amounts of cracks and pinholes, while the film with 3% H_2_O (denoted as perovskite-3% H_2_O) shows a more uniform surface without cracks. A large amount of voids and cracks can be observed when 5% H_2_O (denoted as perovskite-5% H_2_O) was added, which is mainly due to the decomposition of the hydrate perovskite caused by excessive bulk H_2_O [41]. Besides, as shown in the inset of Fig. 3a, the film without H_2_O additive is constructed of random-oriented small crystalline grains with lots of grain boundaries. The grain size of the perovskite-3%H_2_O film is larger than that of the perovskite-5% H_2_O film, though they both exhibit a vertically oriented brick-like morphology. The larger grains in 2D perovskite film results in almost no grain boundary along the vertical direction. It has been reported that grain boundaries are regions where the trap states are mainly distributed [45, 46]. Therefore, the perovskite-3% H_2_O films with larger vertically oriented crystal grains contribute to efficient PVSCs.

The GIWAXS patterns have been used to identify the role of water additive in the crystal growth of 2D perovskite films further. We speculate that water additive can regulate the crystallization process of perovskite because of its lower boiling point and higher vapor pressure compared with DMF [40]. Furthermore, incorporating a suitable amount of water into DMF increases the solubility of perovskite ionic compound, leading to the improved quality of the perovskite films with enhanced crystallinity [47]. The SEM and GIWAXS results in this work are consistent with the speculation. As shown in Fig. 3d, the perovskite-w/o H_2_O film displays several Bragg rings at specific *q* values, indicating mainly random oriented crystal grains within this polycrystalline film. However, the perovskite-3% H_2_O film shows sharp and discrete Bragg spots along the same *q* position (Fig. 3e), which suggests the well-aligned crystal grains with (111) planes parallel to the substrate [17]. Moreover, the darker Bragg spots are observed in perovskite-3% H_2_O film whereas the less apparent diffraction rings in perovskite-w/o H_2_O film, which demonstrates the increased crystallinity of perovskite-3% H_2_O film. The highly oriented perovskite-3% H_2_O film that is perpendicular to the substrate can form an efficient carrier transport channel, leading to improved photovoltaic performance [14, 17].

**Fig. 4:**
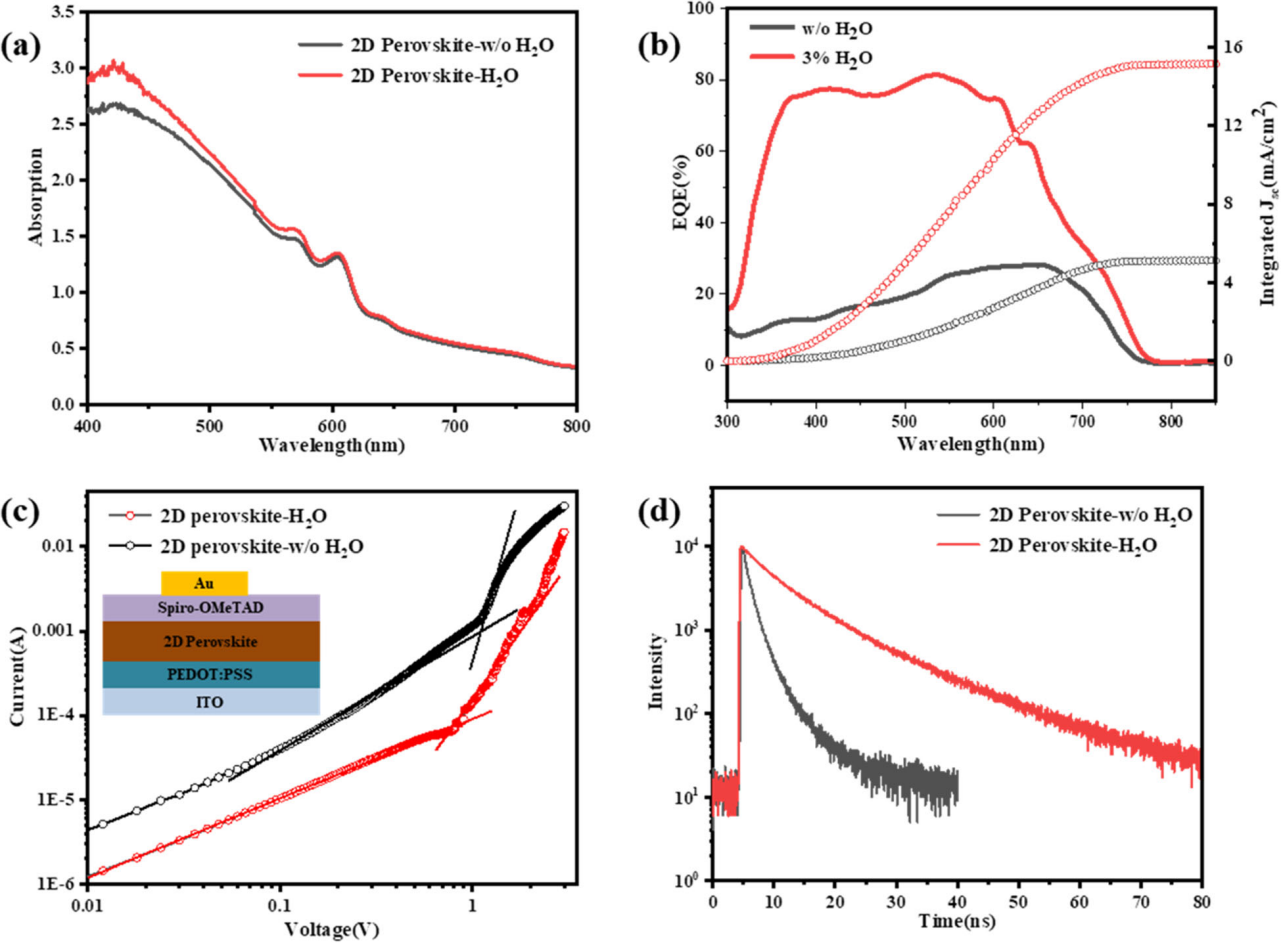
**a** Absorption spectra of BA_2_MA_3_Pb_4_I_13_ films without and with 3% H_2_O. **b** EQE spectra and integrated current curve of corresponding devices. **c** Dark current-voltage curves of the HODs based on corresponding 2D perovskite films (inset: configuration of HODs). **d** TRPL spectra of corresponding 2D perovskite films

To reveal the impact of morphological and crystallographic changes resulted from the addition of water on the optical properties of films, we carried out absorption spectroscopy measurement, as shown in Fig. 4a. Both the perovskite-w/o H_2_O film and the perovskite-3% H_2_O film exhibit multiple exciton absorption peaks in the UV-Vis absorption spectra, indicating the existence of multiple perovskite phases with different *n* values, although nominally prepared as “*n* = 4”. However, the perovskite-3% H_2_O film shows a slightly enhanced absorption in the range of 400-600 nm compared with the perovskite-w/o H_2_O film. From the cross-section SEM images (insets of Fig. 3a-c), it can be concluded that all 2D perovskite films show almost the same thickness. Thus, we attribute the enhanced absorption to a uniform, highly crystalline, and highly oriented perovskite film induced by water additive [14, 48]. The external quantum efficiency (*EQE*) spectrums of PVSC without H_2_O additive and PVSC with 3% H_2_O are shown in Fig. 4b, and the corresponding derived integrated current values are plotted on the right *y*-axis. The integrated *J*_*sc*_ from *EQE* spectrum of PVSC without H_2_O additive and the PVSC with 3%H_2_O is 5.16 mA/cm^2^ and 15.20 mA/cm^2^, respectively. The values are close to the results measured from J–V curve. Apparently, the EQE values of the device with 3% H_2_O in most visible light range are much higher than that of the device without additive. This phenomenon not only results from enhanced light absorption but also mainly comes from more efficient charge transport in highly oriented 2D perovskite film with better crystallinity.

Further, we measured the dark current-voltage curves of the hole-only devices (HODs) with a structure of ITO/PEDOT:PSS/2D perovskite/Spiro-OMeTAD/Au to characterize the trap-state density (*N*_*t*_) in 2D perovskite films (Fig. 4v). The *N*_*t*_ was determined by the trap-filled limit voltage (*V*_*TFL*_) according to equation (1) [14, 46, 49]:

1 $$ {N}_t=\frac{2{\varepsilon}_0{\varepsilon}_r{V}_{TFL}}{q{L}^2} $$

where *ε*_*o*_ is the vacuum permittivity, *ε*_*r*_ is the relative dielectric constant of 2D perovskite, *q* is the elemental charge, and *L* is the thickness of the 2D perovskite film. Both perovskite films have the same *ε*_*r*_ value and the same thickness. Therefore, the *N*_*t*_ is positively correlated with the *V*_*TFL*_ value. As shown in Fig. 4c, the *V*_*TFL*_ value obtained from 2D perovskite-3% H_2_O based HOD is obviously lower than that obtained from 2D perovskite-w/o H_2_O based HOD. It demonstrates that trap-state density in the 2D perovskite-3% H_2_O film has been reduced. This was further confirmed by the time-resolved photoluminescence (TRPL) spectra of the 2D perovskite films deposited on nonconductive glass. The time decay of the fluorescence signals was fitted to two exponentials, as depicted in Fig. 4d. Benefited from high-quality films with few grain boundaries as evidenced in Fig. 2, the 2D perovskite-3% H_2_O film has a longer fluorescence lifetime of 10 ns compared with 2D perovskite-w/o H_2_O film (2 ns), demonstrating the reduced bulk defect density in 2D perovskite-3% H_2_O film.

Based on all above results, we prove that incorporating suitable water additive in precursor solution can control the crystal growth of BA_2_MA_3_Pb_4_I_13_ perovskite film with enlarged grain size and uniform film coverage, leading to a reduced trap-state density. And this highly crystalline and highly oriented BA_2_MA_3_Pb_4_I_13_ perovskite films induced by water additive would facilitate charge transport [8, 9, 14]. Therefore, the high-quality BA_2_MA_3_Pb_4_I_13_ perovskite films bring a comprehensive improvement in *V*_*oc*_, *J*_*sc*_, *FF* of the corresponding PVSCs.

## Conclusion

In conclusion, we have investigated the effects of H_2_O additive on 2D BA_2_MA_3_Pb_4_I_13_ perovskite thin films and the corresponding device performance. By optimizing the amount of H_2_O additive, surface morphology, grain size, and crystallinity of the BA_2_MA_3_Pb_4_I_13_ film are obviously improved and preferred crystalline orientation was obtained. Therefore, optimized 3% H_2_O additive based 2D PVSC yields a significant improvement in PCE from 2.29 to 12.15%. Meanwhile, the shelf stability of the devices is also improved. Our results prove that controlling 2D perovskite crystallization via H_2_O additive is an effective way to obtain efficient and stable 2D PVSCs.

## Data Availability

All the data are fully available without restrictions.

## Abbreviations

2D: Two-dimensional
PCE: Power conversion efficiency
PVSCs: Perovskite solar cells
PEA^+^: Phenethylammonium
BA^+^: Butyl ammonium
H_2_O: Water
NH_4_SCN: Ammonium thiocyanate
NH_4_Cl: Ammonium chloride
DMSO: Dimethyl sulfoxide
TSC: Thiosemicarbazide
MAI: Methyl-ammonium iodide
BAI: n-butylammonium iodide
PC_61_BM: Phenyl-C61-butyric acid methyl ester
BCP: Bathocuproine
spiro-MeOTAD: 2,29,7,79-tetrakis(N,N-di-p-methoxyphenylamine)-9,9-spirobifluorene)
ITO: Indium tin oxide
*J-V*: Current density-voltage
SEM: Scanning electron microscope
GIWAXS: Grazing incidence wide-angle X-ray scatting
*EQE*: External quantum efficiencies
TRPL: Time-resolved photoluminescence
*V*_*oc*_: Circuit voltage
*J*_*sc*_: Short-circuit current density
*FF*: Fill factor
HODs: Hole-only devices
